# Area efficient approximate multiplier based on novel 4:2 compressors and error correction logic

**DOI:** 10.1038/s41598-025-31282-w

**Published:** 2025-12-13

**Authors:** Krishna Prashanth P, Nithish Kumar V

**Affiliations:** School of Electronics Engineering, Vellore Institute of Technology, Vellore, 632014 India

**Keywords:** Approximate computing, Image multiplication, 4:2 Compressor, Error correction logic, Error resilient architecture, Engineering, Mathematics and computing

## Abstract

Multipliers are key components in arithmetic circuits, with their design having a significant impact on overall system performance. Approximate computing techniques seek to improve energy efficiency, processing speed and better use of hardware resources, particularly in applications where that can tolerate minimal accuracy loss. Achieving higher multiplier performance typically requires a careful trade-off between hardware complexity and computational precision. One widely adopted method for designing approximate multipliers involves replacing exact compressors with their approximate counterparts, resulting in a trade-off with accuracy. This paper introduces novel approximate multiplier architectures that partition the computation into three distinct regions: accurate, approximate, and lower region. Partial product compression in the approximate region is carried out using the proposed two 4:2 compressors combined with conventional arithmetic circuits like half adder, full adder and OR logic, to produce the final product. The proposed compressors are developed by analyzing the input occurrence probability of all possible combinations with trade-off between hardware efficiency and computational accuracy. To further improve accuracy, an error correction logic is developed to compensate for inaccuracies in specific input scenarios. Several benchmark error metrics and hardware synthesis using a 32-nm CMOS technology are evaluated for the proposed designs through simulations. Notably, the results of the proposed approximate multipliers shows an average improvements of 70.6% in accuracy, 60.4% in Energy-Delay Product, 30.9% in Power-Delay Product, and 41.6% in delay, outperforming all existing designs considered for comparison. Furthermore, real-time image multiplication experiments were performed using multiple benchmark image datasets, and the output quality was evaluated through the Similarity Index Metric (SSIM) and Peak Signal-to-Noise Ratio (PSNR). In addition, detailed error and heat-map visual analyses were conducted to examine the spatial distribution and intensity of computational errors across pixels. The results demonstrate that the proposed multiplier consistently achieves higher SSIM and PSNR values, along with significantly reduced error concentrations, outperforming existing approximate multiplier designs.

## Introduction

With the widespread use of data intensive applications such as artificial intelligence, machine learning and multimedia there has been significant increase in the energy demand of modern computing platforms. Although transistor scaling continues to reduce costs, while the contemporary devices consume substantially more power and leading to serious design challenges. Furthermore, traditional technology scaling now offers limited improvements, highlighting the need for energy efficient digital circuit design^1^. To mitigate these limitations, approximate computing has been recognized as an efficient design paradigm that capitalizes on the error resilient nature of numerous applications. Approximation techniques can be implemented at various levels of system design, including memory architectures^6^ and arithmetic circuits^2–8^. In arithmetic units, multipliers are the prime element for approximation as they dominate computational cost in digital signal processing (DSP), microprocessors, and neural network accelerators^9^. Conventional multipliers generate partial products through bitwise AND operations and reduce them via multiple level exact compressors and adders, followed by a final carry propagate addition which consumes high power and place an area overheads.

In recent years, the use of approximation in multipliers has emerged as an effective approach to achieving a balance between computational accuracy and hardware efficiency^10^. Lakshmi et al.^11^ reduced accuracy in approximate applications by replacing the lower segment with the more efficient compressor. Momeni et al.^12^ proposed approximate compressors that enhance both delay and energy efficiency in multipliers. Zendegani et al.^13^ developed rounding-based multiplier architectures which reduces power consumption without significantly compromising performance. Akbari et al.^14^ introduced 4:2 compressors capable of switching between approximate and exact modes which provides flexibility depending on application requirements. Shirzadeh et al.^15^ improved output accuracy by selectively controlling specific output bits according to input patterns. Chip-Hong et al.^16^ optimized compressor logic to improve gate-level efficiency, while Moaiyeri et al.^17^ implemented the approximate compressors using basic logic gates. Manikantta et al.^18^ further optimize compressor designs to achieve low error rates. Ansari et al.^19^ extended these optimizations to simultaneously improve speed and accuracy. Venkatachalam et al.^20^ proposed approximate arithmetic multipliers that reduce hardware complexity with trading off some accuracy. Strollo et al.^21^ designed new 4:2 compressors that can be applied either only in the approximate region or across the full multiplier. Vahdat et al.^22^ achieved improvements in delay and power by truncating input operands and ignoring selected partial products. Kong et al.^23^ analyzed delay characteristics of 4:2 compressors, and Liu et al.^24^ used majority-logic compressors to achieve error-resilient designs.

Approximate compressors increase hardware efficiency but inherently introduce errors. To mitigate these, recent studies have integrated error correction blocks with optimized partial product reduction schemes. Strollo et al.^25^ designed low-error 4:2 compressor based multipliers, and Kumar et al.^27^ compensated the errors by replacing the least significant segment with a constant value by applying AND gate based correction for compensation. Haoran Pei et al.^28^ proposed an energy efficient compressors with built-in error correction module. Zakian et al.^29^ and Uppugunduru et al.^30^ introduced correction blocks and algorithmic strategies to reduce partial product errors. Minho et al.^32^ and Mazloum et al.^3^ developed error correction techniques that enhance accuracy with minimal hardware overhead. Vakili et al.^2^ designed Dadda multipliers combining low-power compressors with error-correction modules. Sabetzadeh et al.^33^ achieved designs with improved energy–delay performance. Zakian et al.^29^ implemented 8-bit multipliers using 4:2 compressors with error correction to improve image processing quality. Krishna et al.^34^ introduced low-energy multipliers with novel 4:2 compressors, while Manav et al.^4^ proposed approximate multipliers tailored for edge devices using input-reordered compressors.

Despite these developments, designing approximate multipliers that optimize accuracy, area, delay, and power simultaneously remains challenging. This motivates the development of new architectures that integrate approximate compressors with error correction logic, making full use of application-level error tolerance while preserving hardware efficiency.

Our key contributions in this paper are as follows:

- We proposed probability-driven 4:2 compressors combining hardware efficiency with error resilience.
- Applied truncation or OR logic in the lower region of partial product tree to optimize hardware performance.
- Developed a novel Error Correction Logic (ECL) to match the accuracy of exact multipliers.
- Validated hardware efficiency on 32-nm CMOS technology and evaluated real time application performance through image multiplication.

The rest of the paper is structured as follows: Section 2 details the concept of exact and proposed approximate 4:2 compressors. Section 3 introduces the architectures of the proposed approximate multipliers. Section 4 discusses the comparison of hardware and accuracy metric analysis of the existing and proposed approximate multiplier. Section 5 presents the evaluation of the proposed multiplier for image multiplication applications. Lastly, Section 6 provides a summary of the overall results and discusses the main contributions of the proposed work.

**Figure 1:**
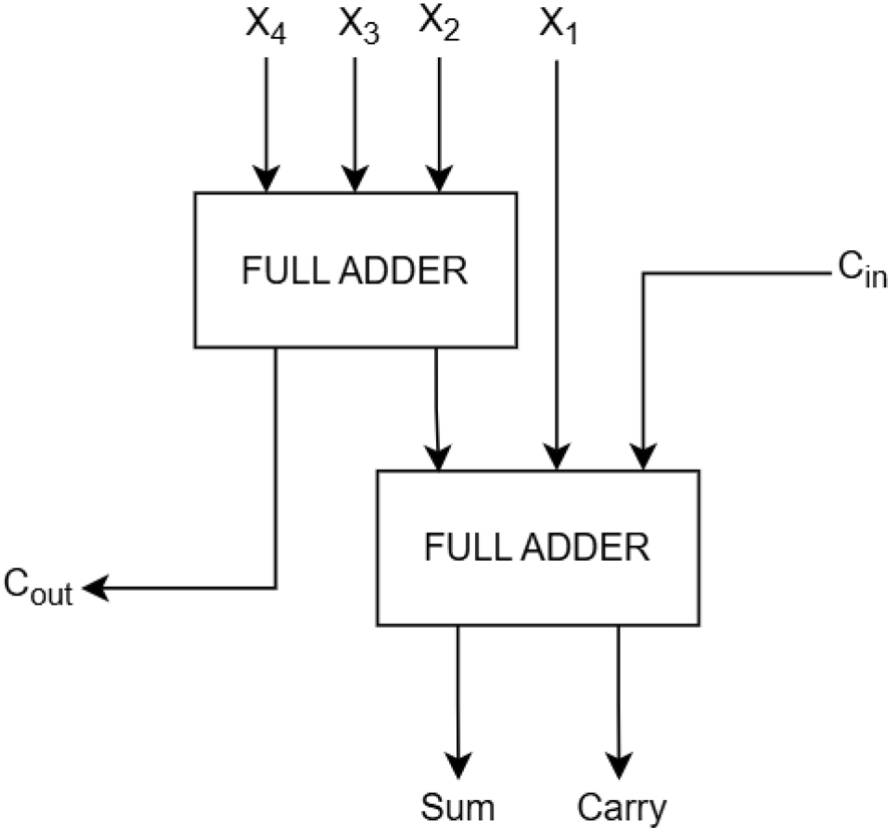
Architecture of exact compressor realized with two full adders^2^.

**Table 1:** Truth table of the exact compressor.

$C_{in}$	$X_4$	$X_3$	$X_2$	$X_1$	$C_{out}$	*Carry*	*Sum*
0	0	0	0	0	0	0	0
0	0	0	0	1	0	0	1
0	0	0	1	0	0	0	1
0	0	0	1	1	0	1	0
0	0	1	0	0	0	0	1
0	0	1	0	1	0	1	0
0	0	1	1	0	0	1	0
0	0	1	1	1	1	0	1
0	1	0	0	0	0	0	1
0	1	0	0	1	0	1	0
0	1	0	1	0	0	1	0
0	1	0	1	1	1	0	1
0	1	1	0	0	0	1	0
0	1	1	0	1	1	0	1
0	1	1	1	0	1	0	1
0	1	1	1	1	1	1	0
1	0	0	0	0	0	0	1
1	0	0	0	1	0	1	0
1	0	0	1	0	0	1	0
1	0	0	1	1	1	0	1
1	0	1	0	0	0	1	0
1	0	1	0	1	1	0	1
1	0	1	1	0	1	0	1
1	0	1	1	1	1	1	0
1	1	0	0	0	0	1	0
1	1	0	0	1	1	0	1
1	1	0	1	0	1	0	1
1	1	0	1	1	1	1	0
1	1	1	0	0	1	0	1
1	1	1	0	1	1	1	0
1	1	1	1	0	1	1	0
1	1	1	1	1	1	1	1

## Exact and proposed approximate 4:2 compressor

Compressor performance has a significant impact on partial product reduction and the overall efficiency of parallel multipliers. This section begins with a review of the exact compressor architecture and then details the design methodologies of the two proposed approximate 4:2 compressors.

### Exact compressor

The architecture of the exact compressor realized using two full adders is shown in Figure 1 . It takes five inputs, namely the four partial product bits $X_4-X_1$ from one column along with an incoming carry $C_{in}$ from the previous column, and reduces them into three outputs: two carry bits ($C_{out}$ ,*Carry*) and one sum bit *Sum* , as summarized in Table 1. The first full adder compresses the inputs $X_4,X_3,X_2$ into an intermediate sum and a carry $C_{out}$. The second full adder then combines this intermediate sum with $X_1$ and the input carry $C_{in}$ to generate the final *Sum* and the second carry *Carry*. The corresponding Boolean expressions for *Sum*, *Carry* and $C_{out}$ in^2^ are are provided as equation  (1) -  (3).

1 $$\begin{aligned} {Sum}= & {\textbf {X}}_4\oplus {\textbf {X}}_3\oplus {\textbf {X}}_2 \oplus {\textbf {X}}_1\oplus {\textbf {C}}_{in} \end{aligned}$$

2 $$\begin{aligned} Carry= & ({\textbf {X}}_4\oplus {\textbf {X}}_3\oplus {\textbf {X}}_2 \oplus {\textbf {X}}_1)\cdot {\textbf {C}}_{in} + \overline{({\textbf {X}}_4\oplus {\textbf {X}}_3\oplus {\textbf {X}}_2 \oplus {\textbf {X}}_1)}\cdot {\textbf {X}}_1 \end{aligned}$$

3 $$\begin{aligned} C_{out}= & ({\textbf {X}}_4\oplus {\textbf {X}}_3)\cdot {\textbf {X}}_2 + \overline{({\textbf {X}}_4 \oplus {\textbf {X}}_3)} \cdot {\textbf {X}}_4 \end{aligned}$$

### Approximate 4:2 compressors

This subsection introduces the structure of the two proposed approximate compressors, designed on the basis of input occurrence probability. In general, approximate compressors are employed in the summation of partial products to achieve reduced power consumption and area overhead while maintaining an acceptable error. Considerable attention in approximate computing has been given to partial product reduction, with various compressors designed to optimize the trade-off between energy use and computational accuracy^2,3^.

**Table 2:** Truth table of existing and proposed approximate 4:2 compressors.

**Input**	**Prob. of**	**[2]**			**[29]**			**PAC1**			**PAC2**		
$x_4 x_3 x_2 x_1$	**each Comb.**	C	S	ED	C	S	ED	C	S	ED	C	S	ED
0000	81/256	0	0	0	0	0	0	0	0	0	0	0	0
0001	27/256	1	0	1	0	1	0	0	1	0	0	0	-1
0010	27/256	0	0	-1	0	1	0	0	1	0	0	0	-1
0011	9/256	1	0	0	0	1	-1	0	1	-1	0	1	-1
0100	27/256	0	0	-1	0	0	1	0	1	0	1	0	1
0101	9/256	1	0	0	0	1	-1	0	1	-1	1	0	0
0110	9/256	0	0	-2	0	1	-1	0	1	-1	1	0	0
0111	3/256	1	0	-1	0	1	0	0	1	-2	1	1	0
1000	27/256	0	0	-1	1	0	-1	1	0	1	1	0	1
1001	9/256	1	0	0	1	0	0	1	1	1	1	0	0
1010	9/256	0	0	-2	1	0	0	1	1	1	1	0	0
1011	3/256	1	0	-1	1	0	-1	1	1	0	1	1	0
1100	9/256	0	0	-2	1	0	0	1	1	1	1	0	0
1101	3/256	1	0	-1	1	0	-1	1	1	0	1	0	-1
1110	3/256	0	0	-3	1	0	-1	1	1	0	1	0	-1
1111	1/256	1	0	-2	1	0	-2	1	1	-1	1	1	-1

**C - Carry, S - Sum and ED - Error distance**

The probability of each partial product combination is determined using the likelihood of an AND gate producing either a ‘1’ or a ‘0’. Since partial products are generated through bitwise AND operations, the output ‘1’ occurs with a probability of $\frac{1}{4}$, while the output ‘0’ occurs with a probability of $\frac{3}{4}$. Based on this distribution, the possible combinations can be categorized into five groups, ranging from the case in which all four outputs are zero ($P_4$) to the case where none are zero ($P_0$). The occurrence probability values for these groups of four input compressor are represented as Equations (4)–(8)^31^.

4 $$\begin{aligned} P_4= & \Pr (x_i y_i)^{4} = 1 \times \left( \frac{3}{4}\right) ^{4} \end{aligned}$$

5 $$\begin{aligned} P_3= & \left( {\begin{array}{c}4\\ 1\end{array}}\right) \, \Pr (x_i y_i)^{3} \, (1 - \Pr (x_i y_i))^{1} = 4 \times \left( \frac{3}{4}\right) ^{3} \times \frac{1}{4} \end{aligned}$$

6 $$\begin{aligned} P_2= & \left( {\begin{array}{c}4\\ 2\end{array}}\right) \, \Pr (x_i y_i)^{2} \, (1 - \Pr (x_i y_i))^{2} = 6 \times \left( \frac{3}{4}\right) ^{2} \times \left( \frac{1}{4}\right) ^{2} \end{aligned}$$

7 $$\begin{aligned} P_1= & \left( {\begin{array}{c}4\\ 1\end{array}}\right) \, \Pr (x_i y_i)^{1} \, (1 - \Pr (x_i y_i))^{3} = 4 \times \left( \frac{3}{4}\right) \times \left( \frac{1}{4}\right) ^{3} \end{aligned}$$

8 $$\begin{aligned} P_0= & (1 - \Pr (x_i y_i))^{4} = \left( \frac{1}{4}\right) ^{4} \end{aligned}$$

Where, the scaling factors 1, 4, 6, 4, and 1, correspond to the occurrence count of the respective input cases for all possible combinations. For one time occurance, the probability for the input pattern ‘1010’ can be computed as the product of the individual input probabilities, i.e., P(1010) = (p($x_4$))(1-p($x_3$))p($x_2$)(1-p($x_1$)) = 9/256. Here, p($x_i$) denotes the probability that input bit being ’1’. Table. 2 shows the probability of occurrence of each combinations and the error distance (ED) measured as the difference in value with exact compressor for each combinations. In our designs, similar to other approaches reported in^2–4,29^, the $C_{in}$ and $C_{out}$ signals are omitted for hardware efficiency considerations. Consequently, the compressor structure produces only the *Sum* and *Carry* outputs.

#### Architecture of the proposed approximate compressors

**Figure 2:**
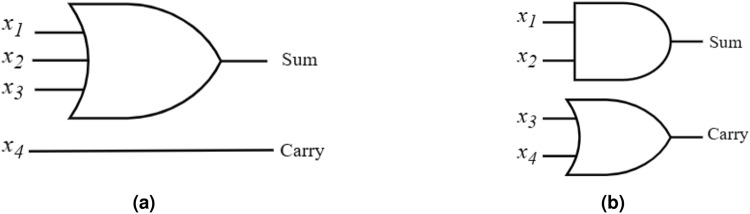
Logical diagram of the two proposed approximate compressors.

In this work, two approximate 4:2 compressor designs are proposed and optimized for reduced hardware. Each compressor accepts four inputs ($x_4$, $x_3$, $x_2$, $x_1$) and produces two outputs (*Carry* and *Sum*), with their corresponding truth table summarized in Table 2. The first design, referred to as Proposed Approximate Compressor-1 (PAC1), is developed by modifying the exact compressor’s truth table according to the probability distribution of its inputs. PAC1 reduces hardware complexity by introducing errors in nine out of sixteen input combinations. The *Carry* output is obtained directly from input $x_4$, while the *Sum* output is computed using a logical OR of the remaining inputs Figure 2a. The Boolean expressions for *Sum* and *Carry* are provided in equations  (9) and  (10). This design achieves lower energy consumption and hardware compared to the exact implementation.

9 $$\begin{aligned} \text {Sum}= & \text {x}_1 + \text {x}_2 + \text {x}_3 \end{aligned}$$

10 $$\begin{aligned} \text {Carry}= & \text {x}_4 \end{aligned}$$

Compared to designs in^2,29^, PAC1 also introduces negative errors for specific input combinations, namely “0011”, “0101”, “0110”, and “0111”. In particular, the error distance reaches -2 for the input “0111”, causing a notable reduction in accuracy. To overcome this limitation, both the input occurrence probabilities and the absolute error distance of each combination are taken into account in the second design. This results in evaluation of Proposed Approximate Compressor-2 (PAC2), which limits the resulting errors to an error distance of either +1 or −1. Moreover, compressors producing errors of opposite signs (e.g., −1 and +1) can partially cancel each other which further reduces the overall error in the multiplier. This design achieves a better balance with power, area and accuracy trade-offs compared to PAC1. The Boolean expressions for *Sum* and *Carry* of PAC2 are provided in equations  (11) and  (12), and its gate-level schematic is shown in Figure 2b.

11 $$\begin{aligned} \text {Sum}= & x_1 \bullet x_2 \end{aligned}$$

12 $$\begin{aligned} \text {Carry}= & \text {x}_3 + \text {x}_4 \end{aligned}$$

## Proposed approximate multipliers architecture

**Figure 3:**
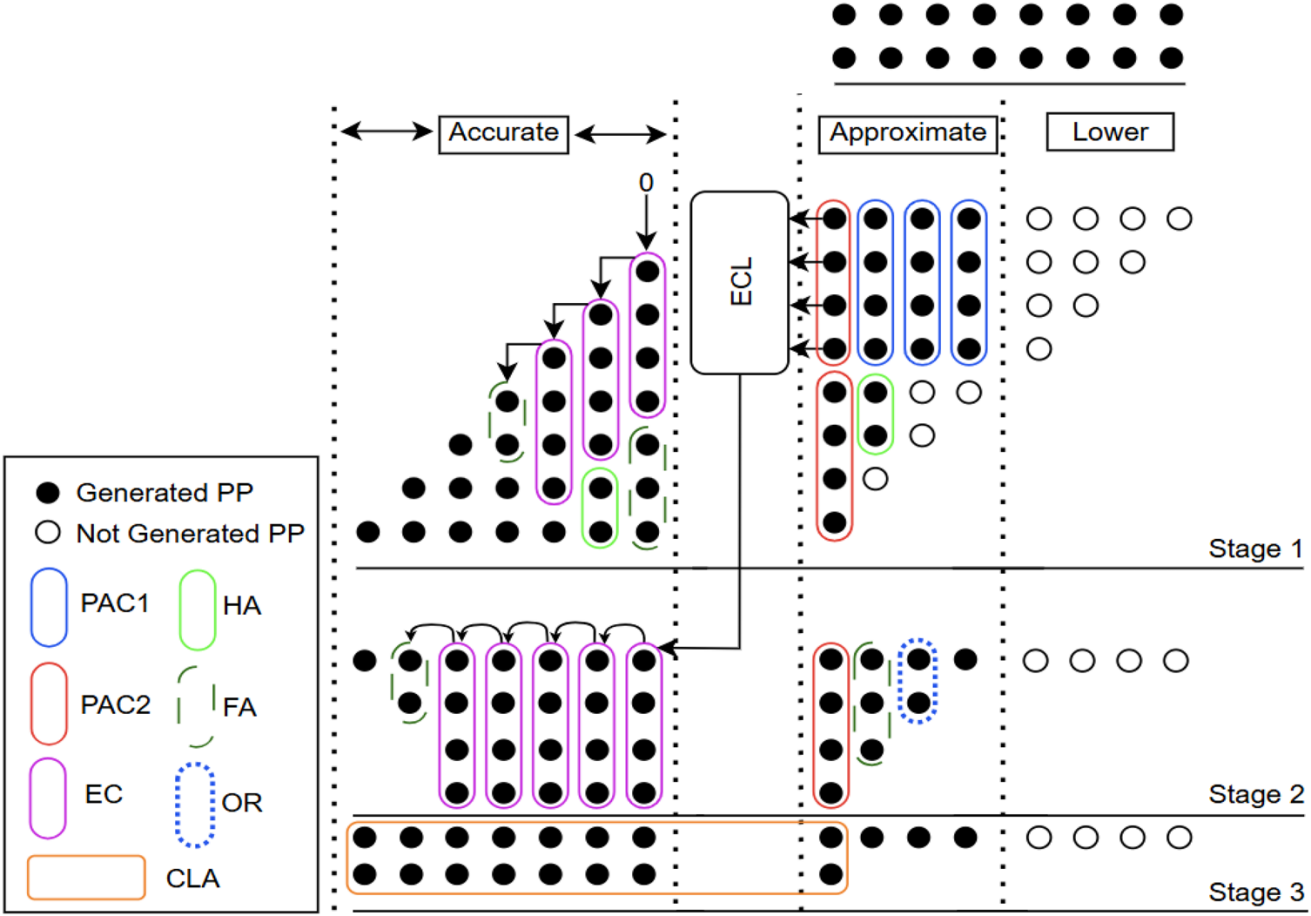
Partial product reduction structure of the proposed approximate multiplier architecture with lower region truncated and employing ECL (AXM2).

Many applications utilize smaller datapath width multipliers, hence the performance of the proposed compressors in arithmetic operations is evaluated on 8-bit unsigned multiplier^35^. In high performance multipliers, the partial product reduction stage is considered the most critical and power demanding component. The multiplication process generally consists of three main stages: (1) partial product generation, (2) reduction of the partial product tree and (3) final carry-propagate addition. Likewise, the partial product tree is segmented into three regions namely, accurate, approximate and lower regions each contributing to the computation of partial products (PPs) as indicated in Stage 1 of Figure 3. In Stage 1, the black dots represent the partial products generated using AND gates, while empty circles denote the omitted partial products that require no hardware.

Generally, introducing errors in the accurate (most significant) region of the multiplier can severely degrade performance. Therefore, all partial product reductions in this region are typically performed using exact compressors (EC) along with conventional half adder (HA) and full adder (FA). Moreover, achieving higher precision at the cost of increased energy consumption is undesirable in approximate computing applications. To balance the precision and performance, in our design EC’s are employed only for the seven most significant columns of the partial product tree. Since partial product reductions in the lower region have minimal impact on the final product, in our approach two techniques are employed on the least four columns to achieve hardware reduction and power efficiency without significant loss in accuracy. However, in the approximate (middle) region i.e., on the remaining four columns the partial product reduction is performed using the proposed approximate compressors further achieves hardware efficiency.

Compared with PAC2, PAC1 is more hardware-efficient as it requires only a 3-input OR gate. In the approximate region of stage 1, three PAC1 are utilized to maximize hardware reduction. Meanwhile, two PAC2 are employed in the last column of approximate region to achieve better error control, as they introduce only +1 or –1 error distances, which naturally tend to cancel each other. To further enhance hardware efficiency, few partial products (PPs) generated by ANDing the least significant bit of the multiplicand are omitted in the approximate region, particularly in Stage 1, as indicated by the empty circles in Figure 3. Comparing to designs in^4,29,30^, only 22 out of the 26 AND gates are required in the Stage 1 approximate region. This approach simplifies Stage 2 as well, where the four compressors used in the approximate region are reduced to a single PAC2 in the eighth column along with adders . In our proposed design, the half adder in the sixth column is further approximated by an OR gate (shown as a dotted circle) to achieve additional hardware reduction. Stage 2 also includes five exact compressors, one half adder (HA), and one full adder (FA) in the accurate region to preserve precision in the most significant bits. By reducing the number of compressors in Stage 2, an 8-bit carry lookahead adder (CLA) can be used instead of the 12-bit CLA in stage 3 which significantly decreasing overall hardware complexity while maintaining accuracy in the final product computation.

**Figure 4:**
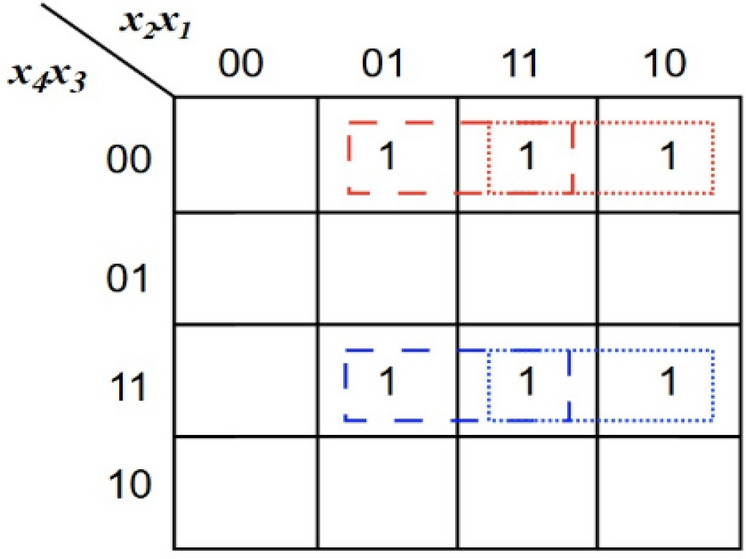
Karnaugh map for the proposed error correction logic (ECL).

**Figure 5:**
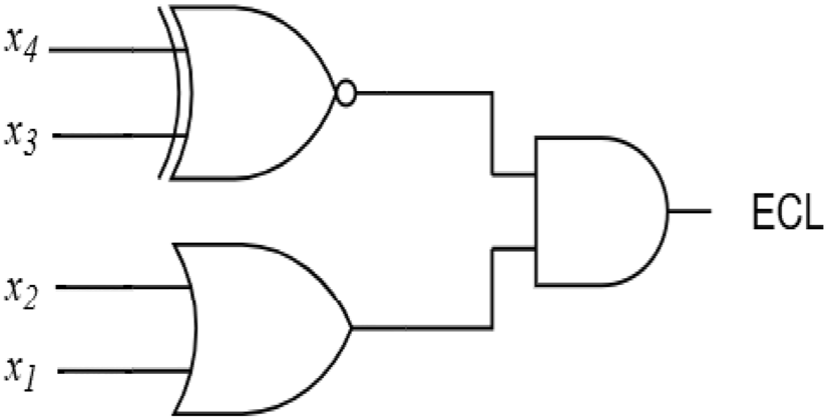
Gate level schematic of the proposed error correction logic (ECL).

Furthermore, to minimize the error propagated to the accurate region, an error correction logic (ECL) is proposed to mitigate the impact of approximation errors. The Karnaugh map shown in Figure 4 represents the Boolean function of $ECL(x_4,x_3,x_2,x_1)$, where the highlighted cells correspond to the input combinations from Table 2, for which the PAC2 generates the negative error distance of ‘-1’. Two groups of minterms are identified: one located in the region where $x_4x_3 = 00$ (red grouping) and another where $x_4x_3 = 11$ (blue grouping). Specifically, for the partial products in column 8, when ‘$x_4x_3$’ is ‘00’ or ‘11’, the ECL should generate a ‘1’, which can be implemented as a XNOR logic between ‘$x_4$’ and ‘$x_3$’. However, this logic additionally introduces a ‘+1’ error for the combinations ‘0000’ and ‘1100’, which may further degrade multiplier performance, especially since the occurrence probability of ‘0000’ is 81/256. Therefore, additional logic must be incorporated alongside the XNOR gate to keep the error distance close to that of the exact compressor. For the other scenarios, when ‘$x_2x_1$’ is ‘01’, ‘10’, or ‘11’, the ECL should generate a ‘1’, but not for ‘00’; this can be implemented as an OR logic between ‘$x_2$’ and ‘$x_1$’. Both groupings span the same adjacent column values 01, 11, and 10, which represent the simplified term $(x_1 + x_2)$. From the K-map grouping, the simplified Boolean expression can be derived as shown in equation (13) and the logical diagram of the proposed ECL is shown in Figure 5.

13 $$\begin{aligned} \begin{aligned} ECL&= \bar{x_4}\bar{x_3}x_1 + \bar{x_4}\bar{x_3}x_2 + x_4x_3x_1 + x_4x_3x_2 \\&= \bar{x_4}\bar{x_3}(x_1 + x_2) + x_4x_3(x_1 + x_2) \\&= (x_1 + x_2) \left( \bar{x_4}\bar{x_3} + x_4x_3 \right) \\&= (x_1 + x_2)\,(x_4 \odot x_3) \end{aligned} \end{aligned}$$

**Figure 6:**
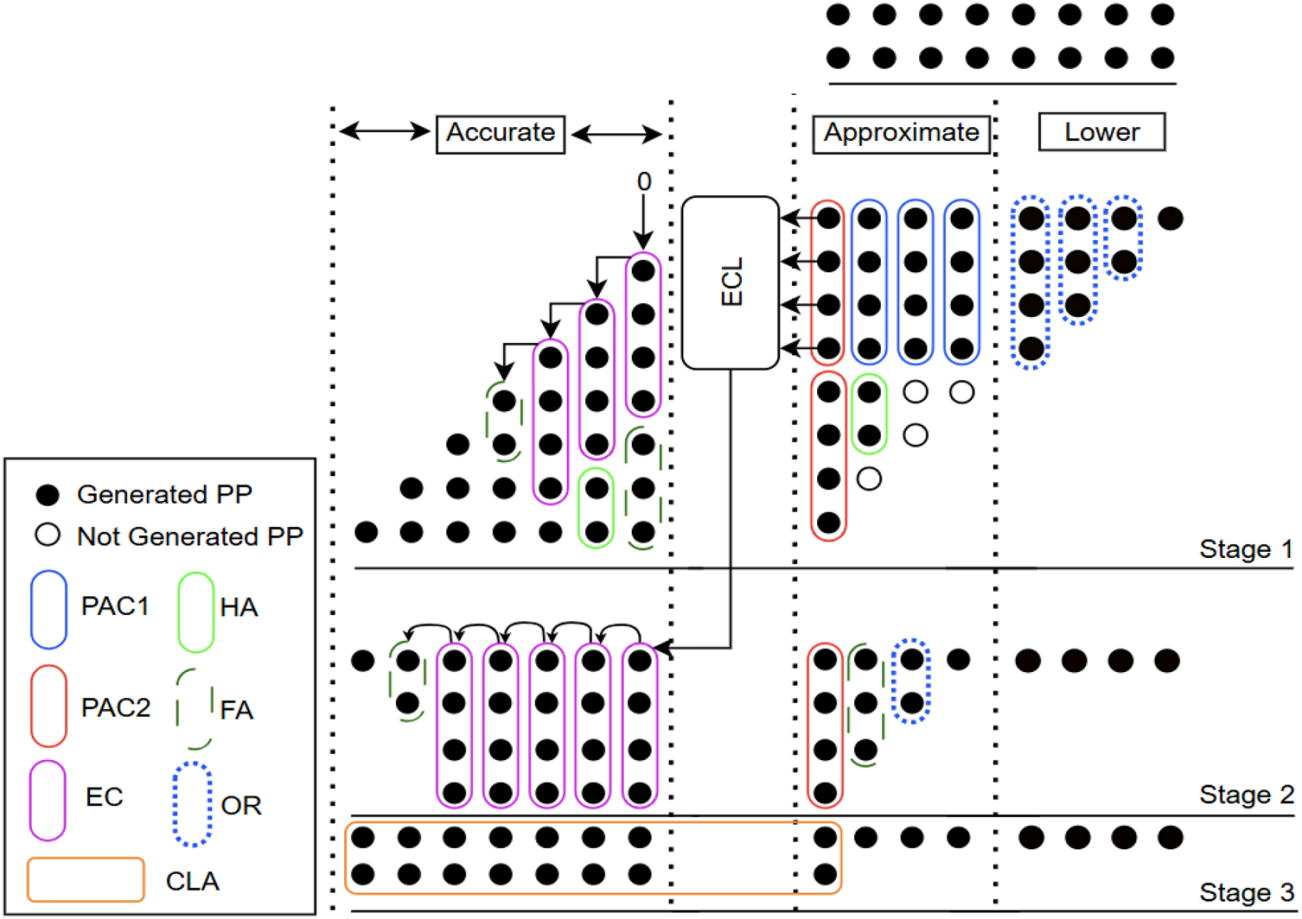
Partial product reduction structure of the proposed approximate multiplier architecture with lower region logically ORed and employing ECL (AXM4).

The four proposed approximate multipliers (AXM1–AXM4) are designed by varying the techniques applied to the lower region and selectively incorporating the ECL, while keeping the approximate and accurate regions unchanged. In AXM1, the lower region is truncated to reduce hardware usage, which introduces errors due to the omittion of least significant bits (LSBs). Specifically, 10 AND gates from the first four columns are removed as indicated on stage 1 of Figure 3.

In AXM2, truncation in the lower region is retained, where the ECL is included to compensate the errors introduced by truncation and improving overall accuracy. AXM3 employs column-wise OR logic in the lower region instead of truncation, which significantly enhances accuracy, with the slight increase in hardware. Finally, AXM4 combines the OR logic approach of AXM3 with ECL, which substantially reduces errors with additional increase in hardware as shown in Figure 6. Collectively, these four architectures provide a flexible design space, offering a favorable trade-off among accuracy and hardware utilization compared to existing multipliers.

## Results and Discussions

In this section, the proposed multiplier architectures are rigorously evaluated and benchmarked against previously reported approximate multipliers. The evaluation highlights the trade-offs between hardware utilization and computational accuracy for the existing and proposed designs.

### Accuracy metrics

To assess the accuracy of the proposed approximate multipliers, several commonly reported metrics were considered, with results obtained through MATLAB simulations . For the 8-bit multiplier, all possible input combinations (i.e. $2^{2N}$ = 65,536 samples) were evaluated, and the results are summarized in Table 3. Metrics such as Mean Error Distance (MED), Mean Relative Error Distance (MRED), Normalized Error Distance (NED), and Number of Effective Bits (NoEB) were computed as given in equations (14) – (17)^36^. The Error Distance (ED) measures the difference between the approximate and exact outputs, while MED represents the average of the absolute error distances across all test cases.

14 $$\begin{aligned} \text {MED} = \frac{1}{2^{2N}} \sum _{i=1}^{2^{2N}} |ED_i| \end{aligned}$$

Here, *N* denotes the number of input samples, and $ED_i$ represents the error distance between the exact and approximate outputs for the $i^{\text {th}}$ input pair. To consider the influence of output values, the Mean Relative Error Distance (MRED) is measured and defined as the average of the absolute error distances normalized with respect to the corresponding exact outputs.

15 $$\begin{aligned} \text {MRED} = \frac{1}{2^{2N}} \sum _{i=1}^{2^{2N}} \frac{|ED_i|}{E_i} \end{aligned}$$

Where, $E_i$ denotes the exact output for the $i^{\text {th}}$ input pair.

The Normalized Error Distance (NED) enables a consistent comparison across multipliers of different sizes by normalizing the Mean Error Distance (MED) relative to the maximum possible output error. For an *n*-bit multiplier, the maximum possible error is $(2^n - 1)^2$ and NED is expressed as:

16 $$\begin{aligned} \text {NED} = \frac{1}{(2^N - 1)^2} \sum _{i=1}^{2^{2N}} \frac{|ED_i|}{2^{2N}} \end{aligned}$$

To assess accuracy with respect to bit significance, the Number of Effective Bits (NoEB) metric is used. It quantifies the count of most significant bits that remain essentially error-free and offering a clear measure of the effective precision of the design.

17 $$\begin{aligned} \text {NoEB} = 2^n - \log _2 (1 + RMSE) \end{aligned}$$

Where, *RMSE* represents the root mean square error of the approximate multiplier.

**Table 3:** Accuracy metrics for the proposed and existing approximate multipliers.

Designs	ER %	MED	NED	MRED	NoEB
PMUL4^2^	98.04	207.19	0.0032	0.0632	7.96
Reorder^4^	98.37	126.13	0.0019	0.0026	8.45
AM8EC-1^29^	96.92	120.54	0.0019	0.0405	8.74
D2^30^	82.32	19.29	0.0002	0.0069	10.98
D4^30^	47.16	10.39	0.0001	0.0023	11.31
MUL4^34^	61.23	38.52	0.0005	0.0060	9.66
MUL2^28^	86.32	147.54	0.0024	0.0891	8.87
Proposed AXM1	94.79	123.44	0.0019	0.0364	8.69
Proposed AXM2	95.62	100.70	0.0015	0.0329	9.05
Proposed AXM3	92.52	119.46	0.0018	0.0338	8.73
Proposed AXM4	91.34	99.80	0.0014	0.0314	9.14

Table 3 shows that the proposed designs employing OR logic in the lower region achieve lower error rates compared to truncated implementations. The improved accuracy of AXM2 and AXM4 can be noticed due to inclusion of the ECL. Specifically, AXM4 demonstrates enhancements of 51.83%, 56.25%, and 50.31% in MED, NED, and MRED, respectively, when compared with PMUL4^2^. Similarly, with respect to AM8EC-1^29^, AXM4 exhibits enhancements of 17.2%, 26.31%, and 4.57%. While the proposed designs show slightly higher errors than the designs^30,34^, this is expected since the compressors in those designs are more hardware intensive. Compared to MUL2^28^, the proposed AXM designs demonstrate substantial accuracy improvement, achieving reductions of up to 31.7% in MED and 2.8$\times$ lower MRED, along with a 10% increase in ER%. For a fair comparison, multipliers^2,29^ using compressors with a comparable error range were considered, as summarized in Table 2. Additionally, the proposed designs achieve superior NoEB values relative to most reported multipliers, with the exception of design D^30^.

Overall, the accuracy analysis demonstrates that AXM2 and AXM4 provide an effective balance between precision and hardware efficiency relative to existing approximate multipliers. Even without error compensation techniques, AXM1 and AXM3 still outperform several of the compared designs in terms of error metrics.

### Hardware synthesis analysis

This subsection discusses the hardware synthesis comparison between the proposed and existing multipliers. All multipliers were described in Verilog HDL and synthesized using the Synopsys Design Compiler targeting a 32-nm CMOS technology. The synthesis environment was configured with the following parameters: a supply voltage of 0.95 V, an operating frequency of 100 MHz, and a temperature of $125 ^\circ$C. In addition, the Gate Equivalent (GE) values are estimated from the synthesized area and normalized using the minimum drive strength 2-input NAND standard cell available in the 32-nm CMOS technology library, ensuring technology independent comparison. It expresses the physical silicon area of a design in terms of the number of 2-input NAND gates. Table 4 summarizes the hardware performance metrics of all multipliers, including area, GE, power, delay, and power–delay product (PDP), which serve as key indicators of hardware efficiency.

**Table 4:** Comparision of hardware performance metrics of proposed and existing designs.

Designs	Area ($\mu m^2$)	GE	Power ($\mu W$)	Delay (*ns*)	PDP (*fj*)
Exact	464.69	304.71	28.76	1.88	54.06
PMUL4^2^	256.73	168.34	14.25	1.31	18.67
Reorder^4^	317.08	207.92	18.02	1.43	25.77
AM8EC-1^29^	378.32	248.07	20.87	1.57	32.76
D2^30^	441.79	289.69	24.11	1.48	35.68
D4^30^	476.52	312.4	24.98	1.55	38.72
MUL4^34^	481.22	315.55	24.72	1.63	40.29
MUL2^28^	404.32	265.12	23.65	1.90	44.94
Proposed AXM1	308.22	202.11	17.27	1.27	21.93
Proposed AXM2	322.02	211.16	18.04	1.29	23.27
Proposed AXM3	331.16	217.15	18.17	1.27	23.07
Proposed AXM4	345.27	226.40	18.94	1.29	24.43

**Figure 7:**
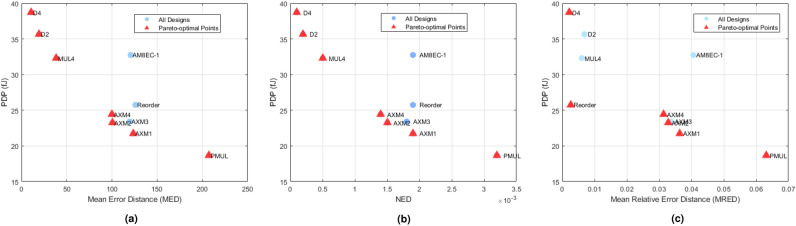
Pareto trade-off plots for various approximate multiplier designs.

In the proposed multiplier variants, the arithmetic structures in the accurate and approximate regions are identical, except for the inclusion or omission of the ECL. Since the carry from the lower region is not propagated to the approximate part in either truncation or ORed logic, the critical path delay remains nearly identical across all variants, as shown in Table 4. The proposed multipliers AXM1–AXM4 achieve lower PDP with acceptable error propagation compared to existing designs^29,30,34^. AXM1 and AXM2 offer the best improvements in area, power, and PDP, with reductions of 33.67%, 40%, and 60%, respectively, relative to the exact multiplier. Among designs with ECL, AXM2 and AXM4 achieve 33.08% and 28.25% area savings and 27.02% and 23.33% power reduction, respectively compared to the best of existing design^34^. Compared to MUL2^28^, the proposed AXM1 design achieves 24% reduction in area, 31% reduction in GE, 37% lower power, 33% improvement in delay, and 2$\times$ better PDP, demonstrating superior PPA efficiency. Based on GE comparison, the proposed AXM multipliers demonstrate significantly reduced hardware complexity up to 30.4% lower than the exact multiplier and consistently smaller than existing approximate multipliers such as AM8EC (248 GE) or D2 (289.69 GE), indicating hardware efficiency and lower implementation cost. AXM1 maximizes hardware efficiency at the expense of accuracy, while AXM4 prioritizes accuracy with higher area and power, suitable for precision-critical applications. AXM2 and AXM3 offer balanced performance, with AXM2 providing higher accuracy than AXM1 and lower hardware overhead than AXM4, making it optimal for area efficient and error-resilient applications with lower PDP.

Additionally,  Figure 7 demonstrates that the proposed multipliers provide an effective balance between power–delay performance and computational accuracy, as indicated by their pareto-efficient positioning when comparing PDP with error metrics including MED, NED, and MRED.

**Figure 8:**
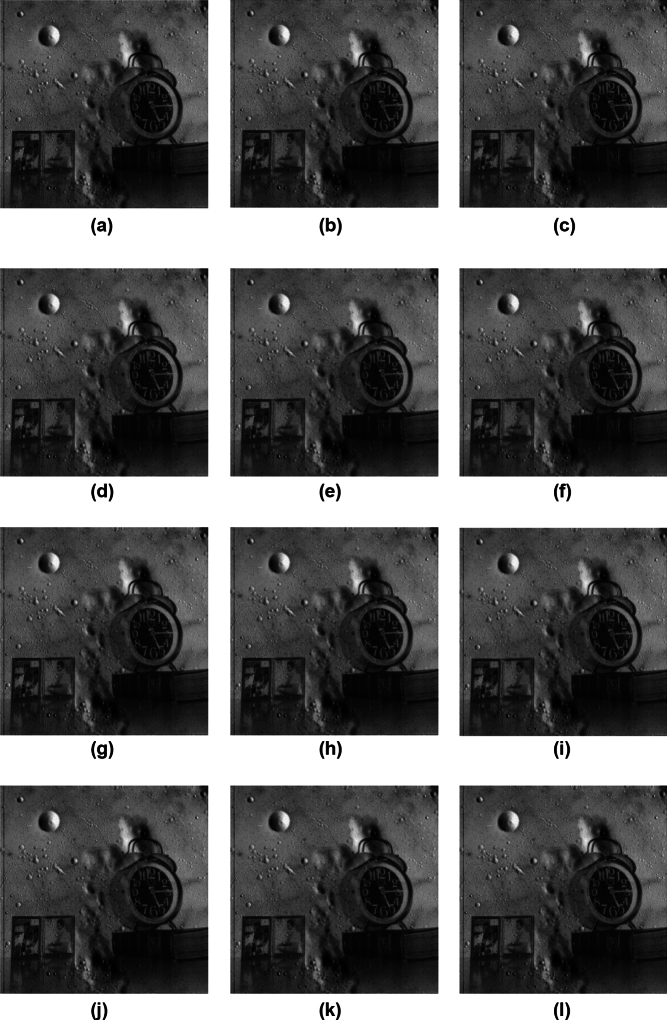
Output of image multiplication on the Moon and Clock images for all proposed and existing multiplier designs.

## Benchmarking application: image multiplication

To demonstrate the practical effectiveness of the proposed approximate multipliers, image multiplication experiments were performed using six standard 256 $\times$ 256 grayscale images such as Airplane, Aerial, Bird, Cameraman, Clock, and Moon. Pixel-by-pixel multiplication was carried out in MATLAB, where each image pair was processed using both exact and approximate multipliers. Figure 8 present a visual illustration of image quality for image multiplication of the Cameraman and Moon images. Quantitative assessment was performed using the Peak Signal-to-Noise Ratio (PSNR) and Mean Structural Similarity Index Measure (MSSIM) and expressed in equations (18) and (19)^37^ .

18 $$\begin{aligned} PSNR = 20log_{10} \left( \frac{Max}{MSE} \right) \end{aligned}$$

Where, *MSE* - Mean Square Error and *Max* - Maximum pixel value of the image, which is 255 for 8-bit grayscale images.

19 $$\begin{aligned} MSSIM = \frac{1}{M}\sum _{i=1}^{M}SSIM(x_{i},y_{i}) \end{aligned}$$

**Figure 9:**
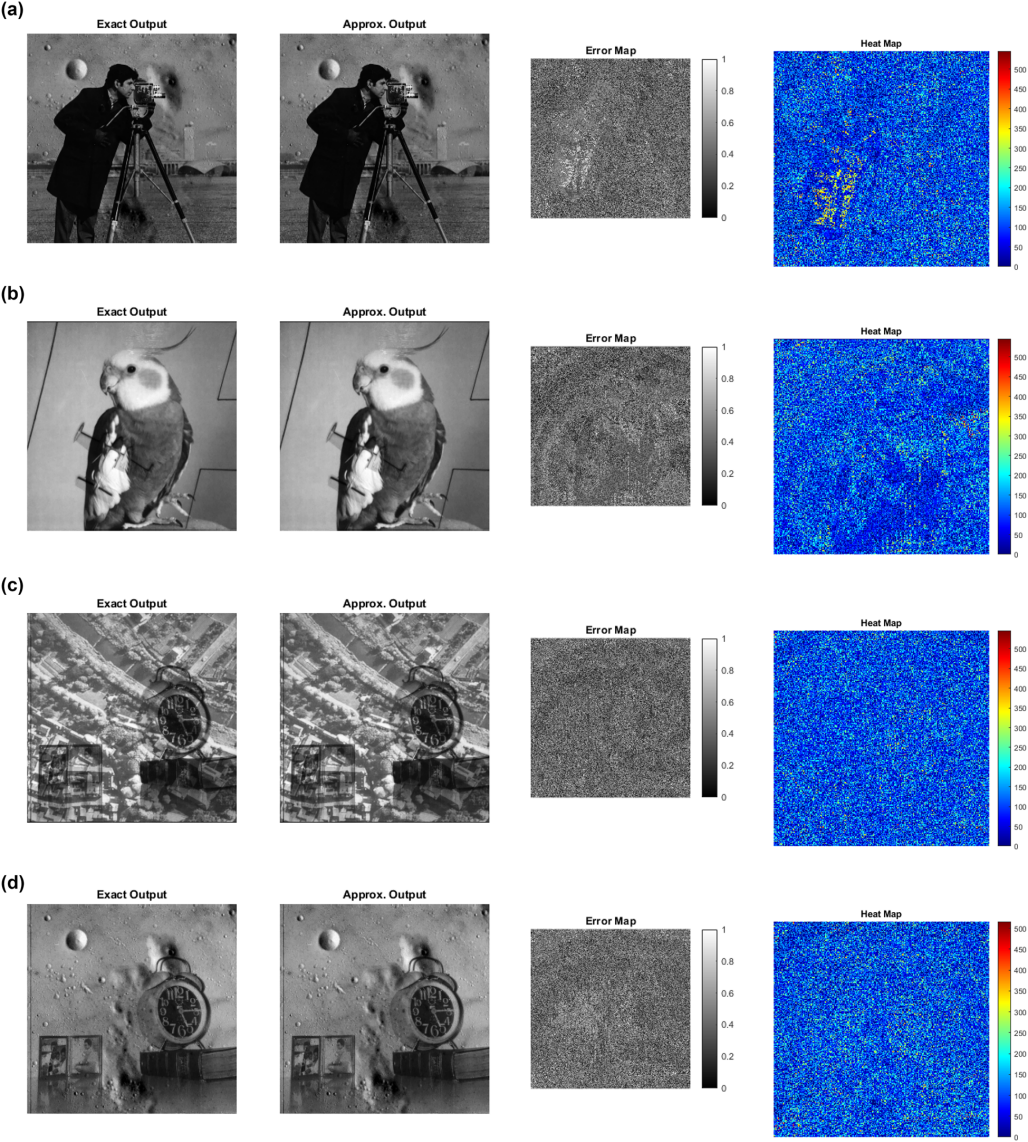
Exact output, Approximate output, Error map and Heat map for (a) Cameraman $\times$ Moon, (b) Airplane $\times$ Bird, (c) Aerial $\times$ Clock and (d) Moon $\times$ Clock.

**Table 5:** Quality metrics comparison between existing and proposed multipliers.

**Designs**	**Cameraman X Moon**		**Airplane X Bird**		**Aerial X Clock**		**Moon X Clock**		**Average**	
	PSNR (dB)	MSSIM	PSNR (dB)	MSSIM	PSNR (dB)	MSSIM	PSNR (dB)	MSSIM	PSNR (dB)	MSSIM
PMUL4^2^	46.80	0.9381	45.72	0.9576	45.97	0.9941	45.75	0.9856	46.06	0.9689
Reorder^4^	48.56	0.9751	49.73	0.9761	50.41	0.9981	50.05	0.9957	49.69	0.9863
AM8EC-1^29^	49.92	0.9783	49.82	0.9752	51.14	0.9980	49.97	0.9958	50.21	0.9868
D2^30^	64.32	0.9990	66.06	0.9995	66.35	0.999	64.22	0.9990	65.24	0.9991
D4^30^	66.45	0.9993	67.18	0.9996	67.54	0.9990	64.49	0.9990	66.42	0.9992
MUL4^34^	58.14	0.9939	58.10	0.9971	52.19	0.9996	58.22	0.9991	56.66	0.9974
MUL2^28^	46.62	0.9211	47.03	0.9372	47.92	0.9530	48.05	0.9647	48.39	0.9698
Proposed AXM1	48.89	0.9650	49.73	0.9761	51.07	0.9984	50.96	0.9964	50.16	0.9840
Proposed AXM2	51.16	0.9689	51.02	0.9803	52.03	0.9986	52.97	0.9967	51.80	0.9861
Proposed AXM3	49.19	0.9691	49.83	0.9766	50.87	0.9984	50.98	0.9964	50.22	0.9851
Proposed AXM4	51.44	0.9695	51.14	0.9808	52.28	0.9986	53.03	0.9967	51.97	0.9864

Table 5 compares the image quality metrics of the proposed and existing multipliers, showing average PSNR and MSSIM values for four benchmark image pairs. Existing multipliers achieve MSSIM values between 0.9689 and 0.9992, and PSNR values ranging from 46.06 dB to 66.42 dB, corresponding to^2^ and D4^30^. The proposed multipliers AXM2 and AXM4 demonstrate higher average PSNR than the reference designs^2,4,29^. Overall, the proposed multipliers achieve an average PSNR of 51 dB and MSSIM of 0.9854, indicating that image quality is largely retained. To further validate the visual fidelity of the proposed approximate multiplier, error maps and heat maps were generated for four standard image pairs and are presented in Figure 9. The error maps is generated through pixel-wise differences between the exact and approximate outputs. Appearing predominantly dark indicates minimal deviation introduced by approximation. Likewise, the corresponding heat maps exhibit mostly low-intensity regions, demonstrating that the approximation introduces only localized and low-magnitude variations. These visual analyses together with PSNR and MSSSIM results confirm that the proposed design achieves high computational accuracy while preserving the perceptual visual quality required for approximate image-processing applications.

## Conclusion and Future work

This paper introduced four variants of area efficient approximate multiplier architectures incorporating two novel 4:2 compressors in the approximation region, with and without error correction logic. The proposed designs significantly improve hardware efficiency while maintaining satisfactory computational accuracy. MATLAB based evaluation demonstrated substantial reductions in error metrics such as MED, NED, and MRED of up to 51.83%, 56.25%, and 50.31%, respectively. Comprehensive error characterization using heat-map and error-map visualization further validated localized error confinement within lower significance regions. Hardware synthesis results confirmed up to 33.6% area savings and 40% reduced power consumption compared to exact designs. The proposed AXM designs outperform the best existing approximate multipliers in the literature, offering up to 35% area, 40% power, and 60% PDP reduction, while maintaining comparable accuracy establishing AXM as the most hardware-efficient architecture. Image multiplication experiments using standard benchmark datasets achieved high PSNR from 50.16% to 51.9 % with consistently high MSSIM values, demonstrating the applicability of the proposed architectures for real-time image processing. The results demonstrate that the proposed AXM multipliers occupy favorable positions along the Pareto front, confirming their superior energy efficiency at comparable accuracy levels. Among all variants, AXM2 exhibits the most balanced performance in terms of accuracy and hardware efficiency, making it suitable for energy-constrained and error-resilient systems.

Future work includes extending the architecture to 16-bit and 32-bit implementations to assess scalability, while preserving the approximate compression and optional error correction strategies and exploring adaptive tunable approximation capable of dynamically adjusting error tolerance based on real-time workloads. Such enhancements would improve suitability for contemporary domains such as edge-AI accelerators, IoT sensor nodes, low-power embedded processors, and real-time image processing platforms.

## Data Availability

The image dataset used in this study was obtained from the publicly available SIPI database (USC, 2022), which is freely accessible online: https://sipi.usc.edu/database/.
